# Chondroblastoma of the Occipital Bone With Atypical Genetic Markers: A Case Report

**DOI:** 10.7759/cureus.22451

**Published:** 2022-02-21

**Authors:** Jessica C Eaton, Bonnie L Cole, Vera Paulson, Hannah E Goldstein, Amy Lee

**Affiliations:** 1 Department of Neurological Surgery, University of Washington, Seattle, USA; 2 Department of Laboratory Medicine and Pathology, University of Washington, Seattle, USA

**Keywords:** pediatric, neuropathology, tumor, occipital bone, chondroblastoma

## Abstract

Chondroblastoma is a rare bone tumor, most often found in epiphyseal plates of long bones. It has infrequently been reported in the skull, most often in the temporal bone. We present a case of chondroblastoma of the occipital bone in a pediatric patient presenting with a bony protuberance of the occiput and imaging consistent with obstructive hydrocephalus, which persisted even after removal of the obstructing tumor. Pathological examination demonstrated that this unusually placed tumor also lacked the classic genetic markers typically associated with chondroblastoma.

## Introduction

Chondroblastoma is a benign bone tumor that most often occurs in the epiphyseal ends of long bones of young males [1]. It is rare, making up only 1% of all bone tumors. Since 1955, fewer than 100 cases of chondroblastoma in the skull have been reported, most often in the temporal bone [2]. On a genetic level, most chondroblastomas are driven by a p.K36M substitution in either *H3F3B* or *H3F3A* [3]. We present a rare case of an occipital chondroblastoma with unique genetic markers requiring postoperative ventriculoperitoneal shunt placement to treat a persistent pseudomeningocele.

## Case presentation

**Figure 1:**
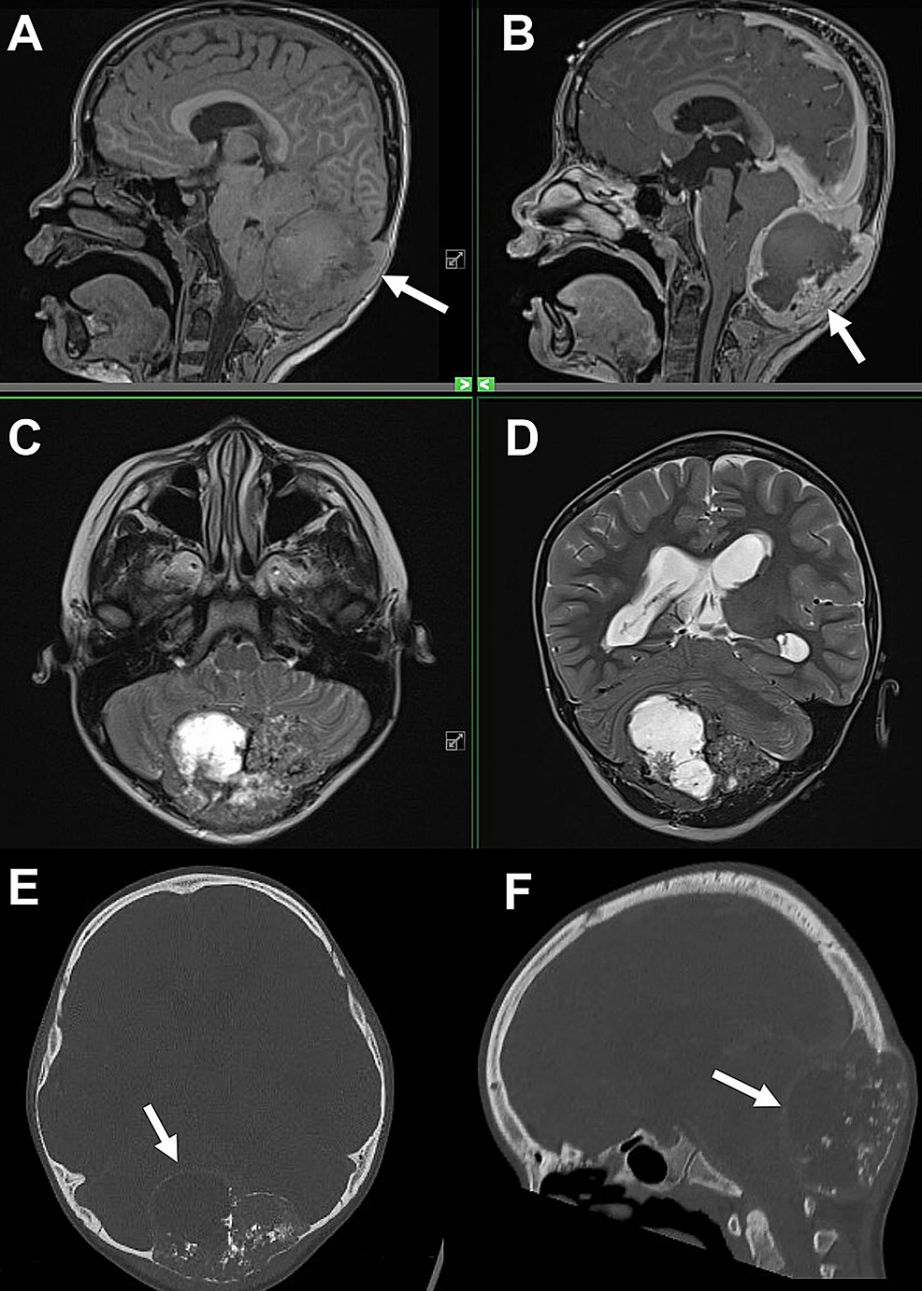
Preoperative magnetic resonance imaging (MRI) and computed tomography (CT) showing lytic mass with cystic components centered in the occipital bone. (A) T1-weighted sagittal MRI showing expansile mass and bony erosion (arrow). (B) T1-weight gadolinium-enhanced sagittal MRI showing peripheral enhancement (arrow). (C) T2-weighted axial MRI showing multilobulated, cystic nature of mass. (D) T2-weighted coronal MRI showing cystic compression of surrounding cerebellum. Non-contrasted head CT demonstrating the lytic nature of the tumor (arrows) in the axial plane (E) and sagittal plane (F).

Our patient was a healthy seven-year-old male who had a several months' history of a bony mass at the occiput. His parents reported that the mass was first noticed after a minor trauma one month prior to his presentation. He was taken to the emergency department for evaluation of the bump when his parents felt that it had increased in size over the preceding weeks. On exam, the bump was firm, non-tender, and non-erythematous. On further questioning about symptoms, the parents reported that his gait had been “off” and he had been urinating more than normal, but he had no headaches or other neurologic deficits. He had no signs of cerebellar-related symptoms and his gait was objectively normal on an exam. Imaging demonstrated a lytic mass with cystic components centered in the occipital bone (Figures 1A-1F). The scan was also significant for compression of the fourth ventricle and associated hydrocephalus. The patient's exam was reassuring, with no signs or symptoms of hydrocephalus.

**Figure 2:**
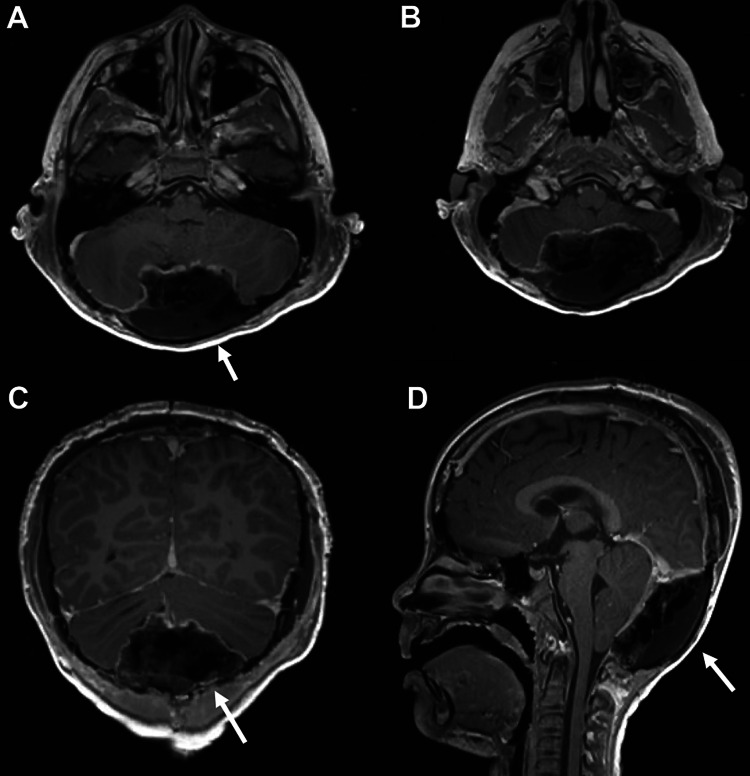
Postoperative magnetic resonance imaging demonstrating gross total resection of the tumor. (A, B) Axial plane. (C) Coronal plane. (D) Sagittal plane.

The patient was taken to the operating room for resection of the lesion. A right frontal extra-ventricular drain (EVD) was placed. The patient was then positioned prone and a bicoronal incision was used to expose the mass. A large, protuberant mass, measuring 5x6x7cm had eroded the bone such that only a thin shell covered the mass. The mass was entirely extradural and was compressing the dura. The tumor was fibrous and very vascular. Deep to the tumor, the dura was intact with the exception of a pinhole-sized partial thickness defect, which was repaired primarily. After resection of the tumor was completed, a parietooccipital craniotomy was performed to obtain a split-thickness bone graft to repair the bony defect at the site of the tumor. A postoperative MRI showed a gross total resection of the lesion (Figures 2A-2D).

Postoperatively, attempts were made to wean the EVD. Unfortunately, the patient developed a persistent pseudomeningocele over the occiput. The decision was made to proceed with a ventriculoperitoneal shunt. After shunt placement, the pseudomeningocele resolved and the patient was discharged home without issue.

**Figure 3:**
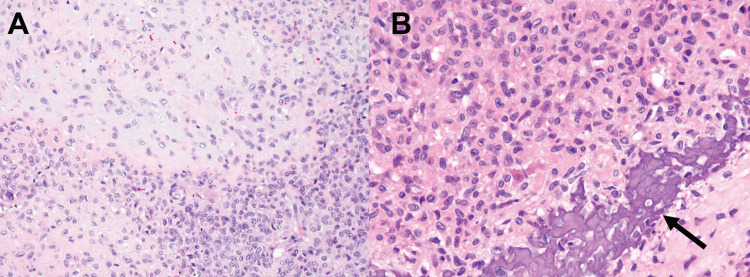
Microscopic images of chondroblastoma and molecular characterization of the tumor sample. (A) 200x H&E stained slide shows the polygonal chondroblasts with zones of cartilaginous matrix between the cells. (B) 400x H&E stained slide shows a typical area of lace-like calcium deposition along the bottom right (arrow).

Pathologic examination revealed a tumor composed of polygonal chondroblasts with a sheet-like pattern of growth interspersed with lobules of amphophilic chondroid matrix (Figure 3A). Interspersed osteoclast-like giant cells were noted along with classic pericellular lace-like calcification (Figure 3B). Occasional mitotic figures were noted. Lesion cells displayed strong nuclear S100 staining by immunohistochemistry, supporting a diagnosis of chondroblastoma.

**Figure 4:**
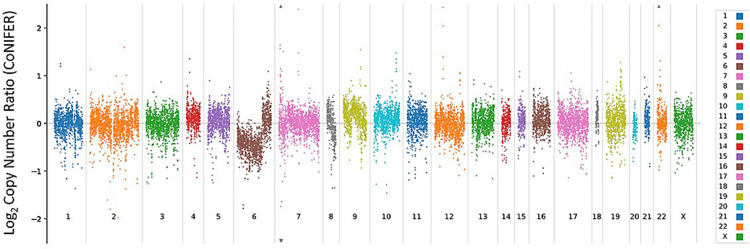
Molecular characterization of the tumor sample. Analysis shows copy loss of a portion of chromosome 6.

Molecular characterization of the tumor was performed using UW-OncoPlex^TM^ version 6 (OPXv6), a targeted next-generation DNA sequencing panel designed to detect single nucleotide variants, insertions and deletions (indels), copy number alterations, and select gene fusions involving 340 genes selected for their importance in the diagnosis, prognosis, and/or treatment of cancer. The platform is also validated to detect microsatellite instability and to assess tumor mutation burden. In brief, total nucleic acid (DNA and RNA) was extracted from formalin-fixed paraffin-embedded (FFPE) tissue using the AllPrep DNA/RNA kit (Qiagen, Valencia, CA); extracted DNA underwent shearing and library preparation followed by sequencing on the Illumina NextSeq500 system (Illumina, San Diego, CA). Sequences were processed through an automated bioinformatics pipeline developed by the University of Washington Next Generation Sequencing Analytics Laboratory, and supplemented by the recent addition of a second copy number caller (CoNIFER), prior to analysis by a board-certified molecular pathologist. The tested tumor sample was found to be positive for copy loss of a portion of chromosome 6 but was otherwise negative for definitive somatic variants, including the classic *H3F3B* and *H3F3A* p.K36M mutations (Figure 4) [3].

## Discussion

Chondroblastomas were first described by Codman in 1931 [1]. Making up 1% of bone tumors, they occur most often in long bones, most frequently affecting young males. Infrequently, chondroblastomas may occur in craniofacial bones or in the skull base. These patients tend to be older, with one report describing five patients aged 39 to 85 years [2], and the most frequent presenting symptom is local swelling, rather than pain [4].

Histopathologically, the chondroblastoma in this report displayed classic microscopic features on pathologic examination. However, the genetic profile of our patient’s tumor showed very atypical markers. The vast majority of chondroblastomas are driven by a p.K36M substitution in either *H3F3B* or *H3F3A*, but neither of these mutations was identified by next-generation sequencing. Additionally, no other molecular drivers such as *IDH1* or *IDH2* alterations were identified. The fact that the classic histone mutations are absent in this case suggests that other genetic alterations may be important in the formation and growth of chondroblastomas.

When the rare skull base chondroblastoma does occur, it is almost always located in the temporal bone. There have been only two previous reports of chondroblastoma of the occipital bone: one in a 16-year-old female [5] and one in a 14-year-old male [4]. The histology of our patient's tumor was consistent with findings described in these previous reports; unfortunately, genetic markers were not available for comparison. Intraoperatively, one patient was found to have a small dural opening where the bony lesion protruded into the posterior fossa. Although he required an initial ventriculostomy, he did not undergo shunt placement.

Our patient also required an initial ventriculostomy because of the obstruction caused by the tumor in the posterior fossa. However, after removing the tumor, the obstruction was no longer present, which was expected to treat the hydrocephalus. Despite multiple attempts to wean the EVD, the patient developed an impressive pseudomeningocele at the surgical site. Although there was no gross violation of the dura in this case, chondroblastomas are known to be highly locally destructive, despite their benign histological nature. In reported temporal bone chondroblastomas, the dura is often involved and requires resection. The patient’s pseudomeningocele may therefore have been attributed to the tumor’s local effect on the dura. While the dura appeared intact, the tumor may have in fact affected the integrity of the dura, allowing the formation of the pseudomeningocele.

## Conclusions

In our patient, a chondroblastoma presenting as a bony protuberance was found to be compressing the fourth ventricle, causing obstructive hydrocephalus. Although the entire tumor was extra-dural, the patient required shunting due to a persistent pseudomeningocele, suggesting that the locally destructive nature of chondroblastoma may have affected the integrity of the dura mater deep to the tumor. Additionally, this tumor was unusual in that it lacked the genetic markers that are typically associated with chondroblastomas. This may suggest that there are other yet undiscovered mutations that may play a role in the formation of chondroblastomas.
